# Supplementary material for: Hierarchical Polyaniline Core-Shell Nanocomposites Coated on Modified Graphite for Improved Electrical Conductivity Performance
Source: Nanomaterials (Basel). 2022 Oct 26;12(21):3776. doi: 10.3390/nano12213776 (PMC9655325; doi:10.3390/nano12213776)
Supplement: Supplementary file 1 [file nanomaterials-12-03776-s001.zip › nanomaterials-1962402-supplementary.pdf]

Supplementary Material

Hierarchical Polyaniline Core-shell Nanocomposites Coated on Modified Graphite for Improved Electrical Conductivity Performance

Asima Naz [1,2], Rabia Sattar [3,*] and Firstname Lastname [2,]

[1] Department of Chemistry, Mirpur University of Science & Technology (MUST), Mirpur, Azad Jammu & Kashmir
[2] Department of Polymer Science & Engineering, Zhejiang University, Hangzhou 310027, China
[3] Department of Chemistry, The University of Lahore, Sargodha Campus, Sargodha, Pakistan
* Correspondence: rabiasattar39@yahoo.com;

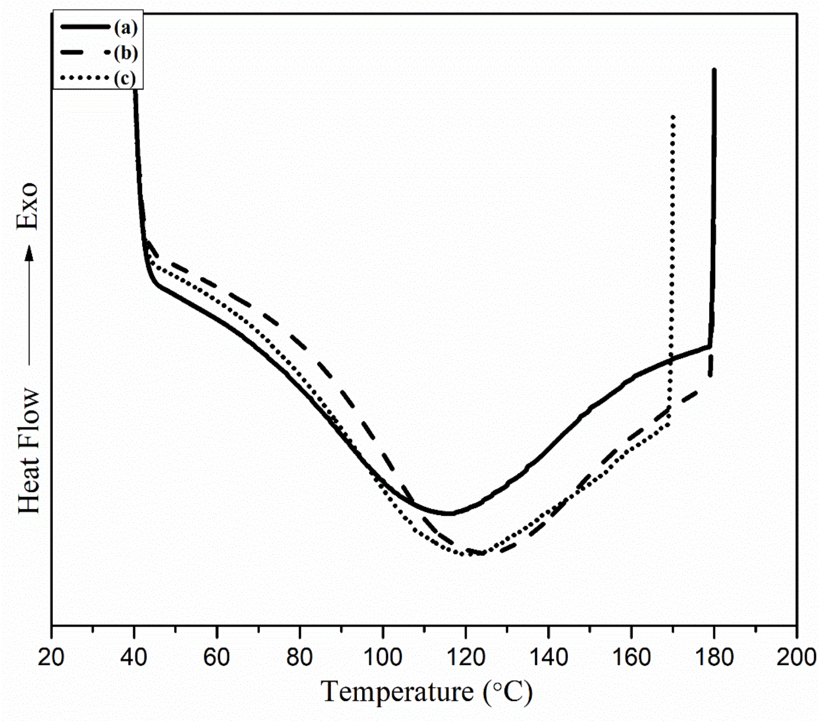

Figure S1. DSC thermograms showing Tg of: **(a)** Composite I; **(b)** Composite II; **(c)** Composite III.

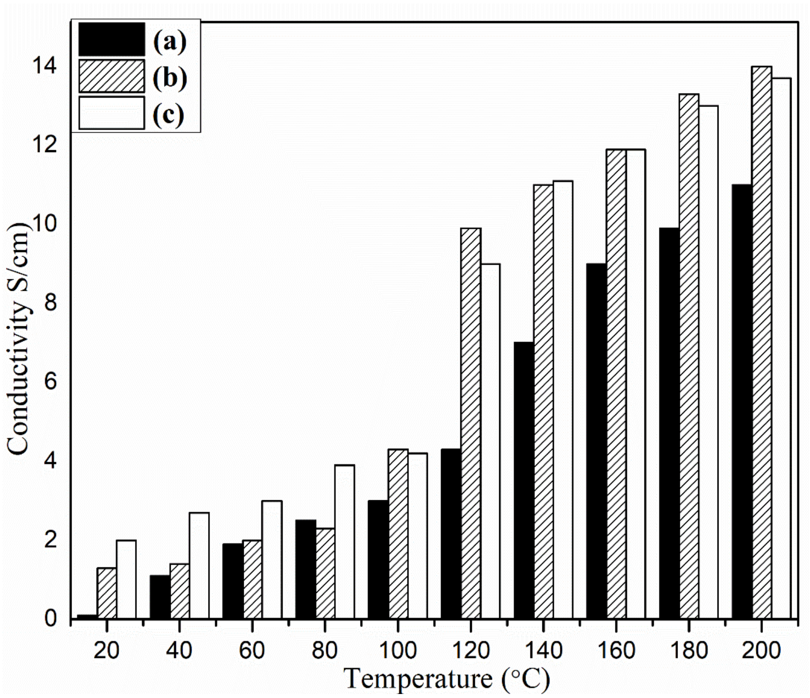

Figure S2. Electrical conductivity display of: **(a)** Composite I; **(b)** Composite II; **(c)** Composite III.

Table S1. FTIR peaks assignment for PANi@FG, PANi/PMMA@FG & PANi/PMMA/PPG-b-PEG-b-PPG@FG.

**Sample**	**υC-O (cm^{-1})**	**υN-H (cm^{-1})**	**υC-N (cm^{-1})**	**ωC-H (cm^{-1})**	**υC=O (cm^{-1})**	**υCH_3 (cm^{-1})**	**Quinoid *vib* (cm^{-1})**	**υ$_{as}$C-H (cm^{-1})**
FG	1050-1412	-	-	2969	1728	-	-	-
PANi@FG	1168	3438, 1167	1398	795	1707	-	1475	2850
PANi/PMMA@FG	1118	3425	1300	663	1721	2921, 1290	1479	2843
PANi/PMMA/PPG-b-PEG-b-PPG@FG	1117	3411	1300	669	1728	-	-	2898

υ = stretch, ω = out-of-plane bending and as = asymmetric

Table S2. TGA data of nanocomposites.

Sample	T_0 (°C)	T_{20} (°C)	T_{max} (°C)	Y_c at 550 °C
PANi@FG	102	351	522	81
PANi/PMMA@FG	111	353	530	82
PANi/PMMA/PPG-b-PEG-b-PPG@FG	113	336	549	71

Table S3. DSC data of PANi/PMMA/PPG-b-PEG-b-PPG@FG composites.

Composite	T_g (°C)	T_m (°C)	T_c (°C)	ΔH_m (J g^{-1})	ΔH_c (J g^{-1})
PANi@FG	67, 114	143	173	0.403	0.793
PANi/PMMA@FG	74, 124	145	174	0.449	1.22
PANi/PMMA/PPG-b-PEG-b-PPG@FG	103, 120	160	178	1.02	2.06

Table S4. XRD parameters showing relative peak position of prepared multilayered nanocomposites.

Sample	Peak position	FWHM	d-spacing
PANi@FG	26.55	0.433	4.25
PANi/PMMA@FG	26.51	0.47	4.46
PANi/PMMA/PPG-b-PEG-b-PPG@FG	26.50	0.31	4.47
Composite I	26.65	0.47	4.43
Composite II	26.59	0.47	4.44
Composite III	26.53	0.47	4.46

Table S5. Textural information of PANi@FG, PANi/PMMA@FG & PANi/PMMA/PPG-b-PEG-b-PPG@FG.

Material	S_{BET} (m^2 g^{-1})	D_p (nm)	V_p (cm^3 g^{-1})
PANi@FG	0.554	3.43	3.18
PANi/PMMA@FG	1.85	4.60	1.27
PANi/PMMA/PPG-b-PEG-b-PPG@FG	4.58	2.27	5.19

V_P: Pore volume; D_p: Pore size distribution; S_{BET}: BET surface area
